# Do zoo‐housed primates retreat from crowds? A simple study of five primate species

**DOI:** 10.1002/ajp.23386

**Published:** 2022-04-29

**Authors:** Alexandria Cairo‐Evans, Natasha K. Wierzal, Jason D. Wark, Katherine A. Cronin

**Affiliations:** ^1^ Division of the Social Sciences University of Chicago Chicago Illinois USA; ^2^ Animal Welfare Science Program, Lincoln Park Zoo Chicago Illinois USA

**Keywords:** animal welfare, monkeys, space use, visitor effects, ZooMonitor

## Abstract

An animal's welfare state is directly influenced by the mental state, which is shaped by experiences within the environment throughout the animal's life. For zoo‐housed animals, visitors to the zoo are a large part of that environment and a fluctuating influence within it. This study examines the impact of zoo visitors on the space use of five species of zoo‐housed primates (Eastern black‐and‐white colobus monkeys, *Colobus guereza*, *n* = 5, Allen's swamp monkeys, *Allenopithecus nigroviridis*, *n* = 2, DeBrazza's monkeys, *Cercopithecus neglectus*, *n* = 3, Bolivian gray titi monkeys, *Callicebus donacophilus*, *n* = 3, and crowned lemurs, *Eulemur coronatus*, *n* = 3). Specifically, we considered whether primates' distance from visitor areas changed as crowd sizes increased. Data were collected using the ZooMonitor app. Observers recorded spatial coordinates for each animal over periods ranging from 12 to 32 months. Data were analyzed using two types of regression models (linear and logistic) to examine the influence of visitors on the location of the primates. Both analyses revealed a statistically significant but small *decrease* in primate distance from visitor viewing glass as the number of visitors increased. Behavioral indicators of welfare were also unaffected by the presence of visitors. These results suggest that, with additional validation, distance from visitors may be one promising, simple way to evaluate the influence of visitors on primate welfare.

## Research Highlights

- The relationship between zoo crowd size and distance from visitors was examined for five primate species.
- Primates did not retreat as crowd sizes increased.
- Primates showed a small but significant decrease in distance from increasing crowd sizes.
- Proximity to visitors may be valuable as a simple tool for inferring animal welfare.

## Graphical abstract

Five species were studied in their zoo habitats. None showed an increase in distance from viewing windows as crowds increased.^*^

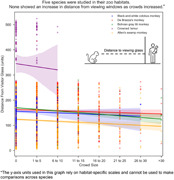

## 1 INTRODUCTION

How animal behavior is impacted by the presence of visitors is a primary question in the study of zoo animal welfare (e.g., Binding et al., 2020; Davey, 2007; Rose et al., 2020; Sherwen & Hemsworth, 2019). A dominant framework for assessing animal welfare is the five domains model (Mellor & Reid, 1994; Mellor et al., 2020). In this model, the four domains of nutrition, environment, physical health, and behavior, impact welfare by influencing the fifth domain, the animal's mental state. This approach emphasizes the individual animal's internal and affective response as key to determining an animal's welfare state. Most studies that attempt to characterize zoo and aquarium animal welfare do so by measuring animal behavior to infer welfare state (Binding et al., 2020).

The conclusions researchers have drawn about the impact of visitors on the welfare of zoo‐housed animals vary (Boyle et al., 2020; Davey, 2007; Hashmi & Sullivan, 2020; Hosey et al., 2020; Miller et al., 2021; Sherwen & Hemsworth, 2019). Some have reported a negative influence of visitors on behavioral indicators of welfare (e.g., Das Gupta et al., 2017; Fernandez et al., 2009), others reported no effect (e.g., Collins et al., 2017; Hansen et al., 2020; Smith & Kuhar, 2010), and still others a positive effect (Claxton, 2011; Jones et al., 2016). Further complicating matters, when comparing studies in which the same behaviors occurred, such as an increase in activity, conclusions about whether these behaviors indicate a positive or negative change with regard to animal welfare have been debated (Choo et al., 2011; Watters et al., 2021; Wood, 1998). For a comprehensive review and interpretation of the influence of visitors on animals, see Sherwen and Hemsworth (2019).

Here, we study the effects of zoo visitors on animals by exploring how the presence of visitors may influence the ways in which primates use available exhibit space. Specifically, we consider whether primates, dependent upon the number of visitors present, increase their distance from the viewing glass. We regard animal location relative to visitors as a simple measure to determine how visitors impact animal welfare (Bonnie et al., 2016; Ross et al., 2009). With full access to all parts of the exhibit, where an animal chooses to be at any point in time may be an indication of where that animal is most comfortable given the current circumstances of the environment and the animal's internal state (Costa et al., 2018; Ramont et al., 2021a; Ross et al., 2009). In efforts to avoid environmental variables that are likely to cause psychological discomfort, animals may move away from what would otherwise cause a negative mental state. A similar phenomenon, the flight initiation distance, has been well studied in birds (Ydenberg & Dill, 1986) to reflect the perceived risks associated with a stimulus or predator. Considering animal distance from guests to study the influence of visitors on primate welfare has been used at least once previously by Bonnie et al. (2016), who measured whether gorillas and chimpanzees occupied areas near the viewing glass more or less than expected by chance as the number of visitors changed. They found no effect of visitors on the primates' tendency to be located near the glass and concluded that the animals were not negatively influenced by the presence of guests, considering exhibit design features as a potential influence on their findings.

We utilize animal proximity to zoo visitors as a simple and potentially decisive tool for inferring animal welfare via affective state. One benefit of this measure is that it can be sampled across a wide range of species and habitats, and calculating the relationship between proximity and visitor presence is relatively straightforward. Additionally, the simple training required to effectively record location data, particularly when compared to more complex behavioral metrics, should increase accessibility and allow broad adoption of this tool across many facilities. This simple method can be augmented by further analyses including more traditional measures such as behavior. Even in the absence of additional measures, we are optimistic that examination of the relationship between animal location and visitor presence may provide valuable initial insights into internal states and potential visitor impacts on the animals' behavior and choices.

In the present study, location data indicating the distance of five primate species from visitor viewing glass is analyzed with respect to the number of visitors present. We consider both how distance is affected by increasing visitor numbers (following Todd et al., 2007; Woods et al., 2019) and how distance is affected by the complete absence of visitors (following Hutchings & Mitchell, 2003; Learmonth et al., 2018; Sherwen et al., 2015). Our goal was to understand how visitors may impact where animals choose to be located in their habitat and specifically whether primates avoid visitors, which may indicate a detrimental impact on animals' internal state and, subsequently, welfare. To accomplish this, we utilized a long‐term data set collected as part of an ongoing behavioral monitoring program at Lincoln Park Zoo (Wark et al., 2019).

## 2 METHODS

### 2.1 Study site and subjects

**Figure 1:**
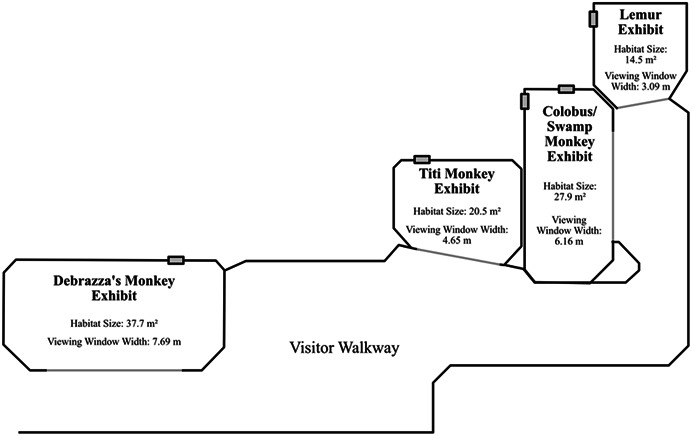
The layout of the four primate exhibits housing study subjects. For each exhibit, the exhibit size (i.e., floor area) and the width of the viewing window are noted.

**Figure 2:**
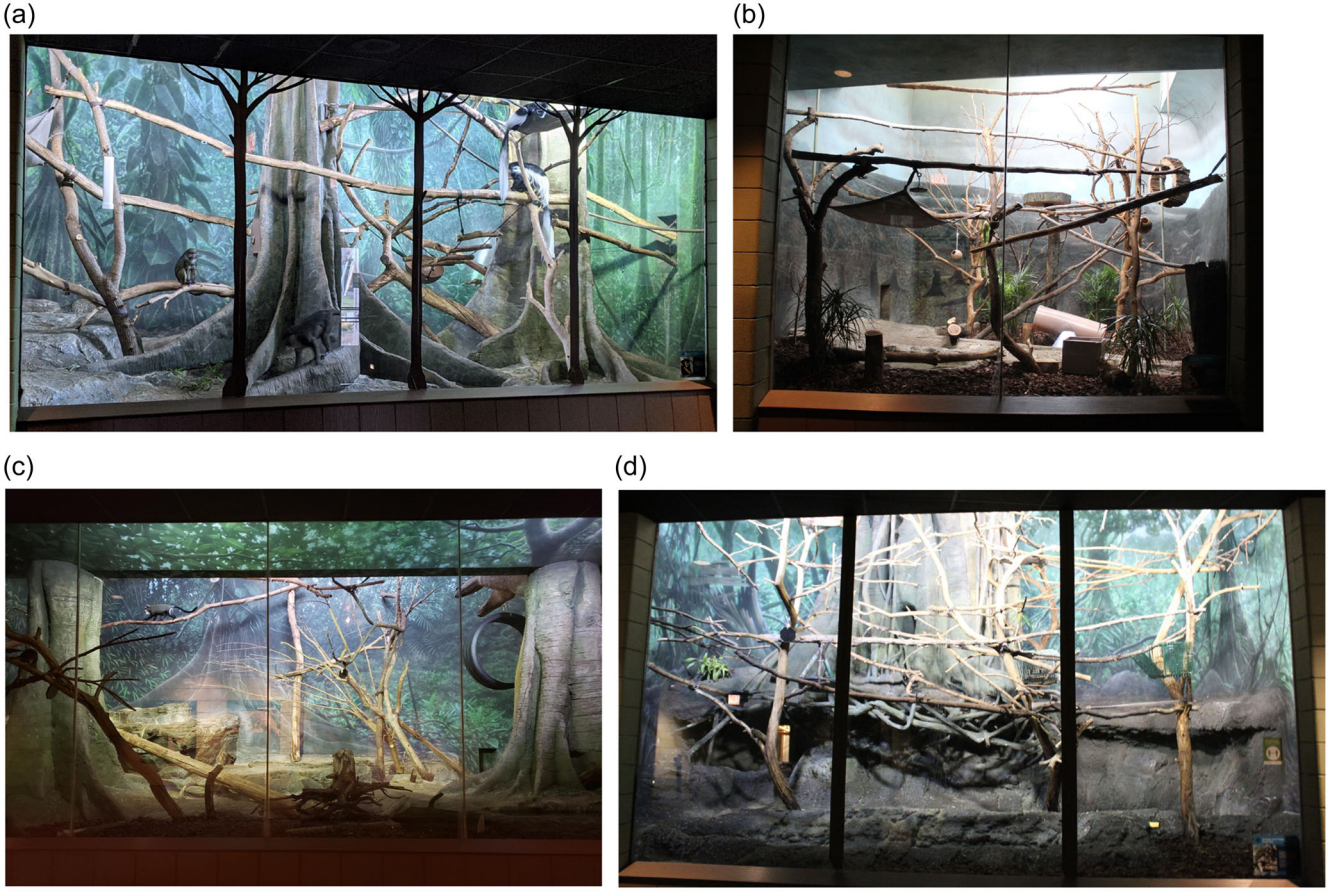
Photographs of the (a) shared exhibit of the Allen's swamp monkey and eastern black‐and‐white colobus monkey, (b) crowned lemur exhibit, (c) Debrazza's monkey exhibit, and (d) Bolivian gray titi monkey exhibit. These photos are taken front‐on from the visitor viewing area.

This study involved four indoor exhibits at the Helen Brach Primate House at Lincoln Park Zoo in Chicago, IL. Data were collected on 5 species and 16 individuals (Table 1, Figure 1). The black‐and‐white colobus monkeys and the Allen's swamp monkeys shared the same exhibit; all other species inhabited their own exhibit. All individuals in this study were captive‐born. The four exhibits vary in size (Figure 1), and photographs of each of the habitats studied here are shown in Figure 2. Habitats were all located in one building and had slightly different environmental features (Figure 4).

**Table 1:** Subject details by species name including individuals' sex, birthdate, number of observations used in this study, and the dates corresponding with the beginning and end of the observation period for each subject.

Subject	Sex	Birthdate (M/D/Y)	Number of observations	Observation start date	Observation end date
**Eastern black‐and‐white colobus**			**224**		
Bea	M	11/13/1993	47	10/6/2019	a10/25/2020
E.B.	F	12/252016	50	10/6/2019	a10/25/2020
Ruk	F	1/252018	41	10/6/2019	a10/25/2020
Nol	F	12/22/2018	38	10/6/2019	a10/25/2020
Kut	F	3/15/2004	48	10/6/2019	a10/25/2020
**DeBrazza's**			**1816**		
Myl	M	9/20/2000	738	1/18/2018	a10/29/2020
Ros	F	6/22/2013	768	1/18/2018	a10/29/2020
Bom	M	11/9/2018	310	2/14/2019	a10/29/2020
**Bolivian gray Titi**			**2091**		
Del	F	5/15/1991	697	7/24/2017	3/12/2020
Och	M	10/17/2011	687	7/24/2017	3/12/2020
Oca	M	3/27/1997	707	7/24/2017	3/12/2020
**Crowned lemur**			**131**		
Sok	M	5/18/2007	44	10/6/2019	3/12/2020
Tsi	M	4/15/2017	43	10/6/2019	3/12/2020
Len	M	4/17/2016	44	10/6/2019	3/12/2020
**Allen's swamp**			**1257**		
Kid	F	6/23/2008	635	6/8/2018	a3/5/2021
Bok	M	10/4/2006	622	6/8/2018	a3/5/2021

^a^ No observations took place between 3/13/2020 and 8/12/2020.

**Figure 3:**
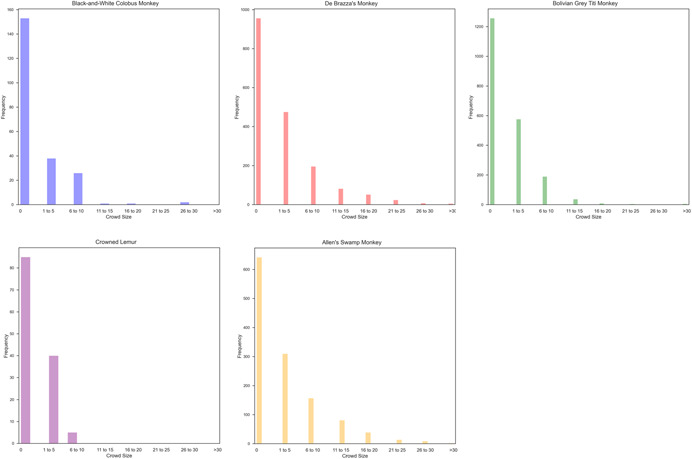
Distribution graphs for each species showing the frequency at which the number of visitors recorded during an observation window totaled 0, 1–5, 6–10, 11–15, 16–20, 21–25, 26–30, or >30, corresponding with the categorical bins used in analyses.

Animals had rare access to out‐of‐view, off‐exhibit spaces intermittently throughout the study. It is possible but not likely that animals could be out of view at times other than while off‐exhibit, so we opted to exclude all cases in which subjects were recorded as out of view. We characterize it as unlikely that animals were on exhibit but out of view because observers were instructed to walk across the length of the viewing glass and look up into the exhibit. With observations recorded in this way, there are very few possible spots for an animal to fully hide. More common are instances of obstructed view of subjects, for which location is still recorded (Figure 3).

Visitor viewing glass panels in each exhibit were transparent on both sides, with the interior habitat more brightly lit than the visitor area, and no additional fence or barrier present to separate animals from guests. Previous audio recordings in one of the study habitats (Bolivian gray titi monkeys) during an evening event indicated that sound levels in habitats were largely independent of the sound levels from the public floor as a result of attenuation from the exhibit glass as a barrier (unpublished data).

### 2.2 Data collection

Space use data were collected as part of routine behavioral monitoring by trained volunteers using Apple® iPads® and the ZooMonitor application developed by Lincoln Park Zoo (2020). All observers met the criteria of 85% interobserver reliability on behavior (percent agreement) and location (percent agreement based on a 30‐pixel space use threshold) with an experienced observer (Wark et al., 2019).

Observations took place between 10:00 a.m. and 4:00 p.m. up to 7 days per week, from July 2017 through October 2020. Thirty trained observers contributed data to this study. Some data collection occurred while the zoo was entirely closed to visitors during the COVID‐19 pandemic. Exact observation date windows per species are available in Table 1.

Observers selected a focal subject to observe based on a randomized schedule. The number of visitors present was recorded immediately before starting an observation session. The maximum number of visitors and frequency of visitor counts at each habitat varied, and is shown in Figure 3. The focal animal location was recorded during 10‐min observation sessions at 1‐min intervals. The ongoing data collection protocol included information on behavior and course and categorical information on height (low, medium, and high). We did not utilize height data in the present study due to between‐habitat differences in height designations and because both within and between habitats the available options to inhabit those zones differed (e.g., based on the presence or absence of branches to perch on).

**Figure 4:**
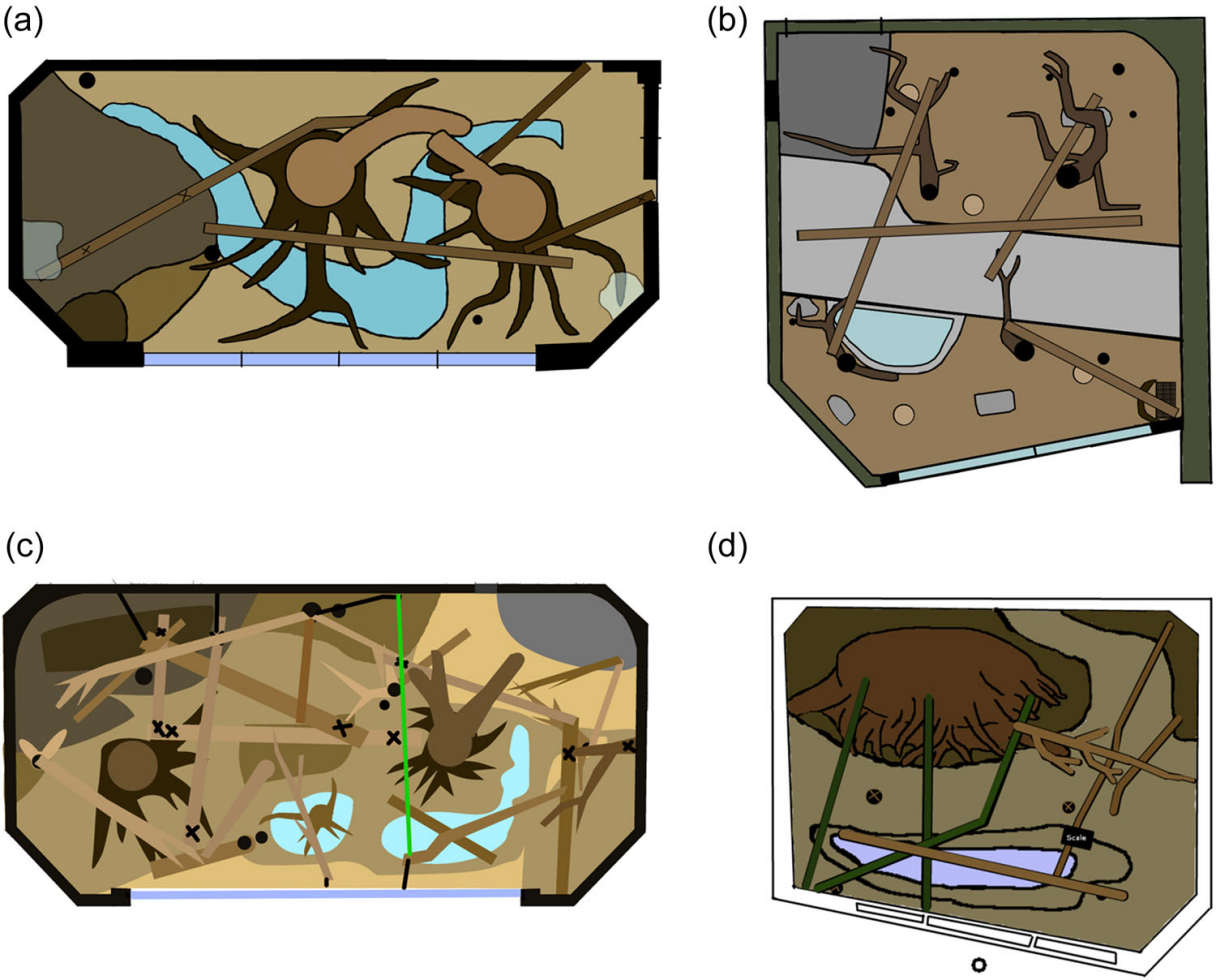
The 2‐dimensional habitat maps of the (a) shared exhibit of the Allen's swamp monkey and black‐and‐white colobus monkey, (b) crowned lemur exhibit, (c) Debrazza's monkey exhibit, and (d) Bolivian gray titi monkey exhibit. These exhibit maps were uploaded to the ZooMonitor app to record space use locations of the monkeys during behavior observations. The maps are from a top‐down perspective, showing major pools, trees, and branches available within the exhibit.

**Figure 5:**
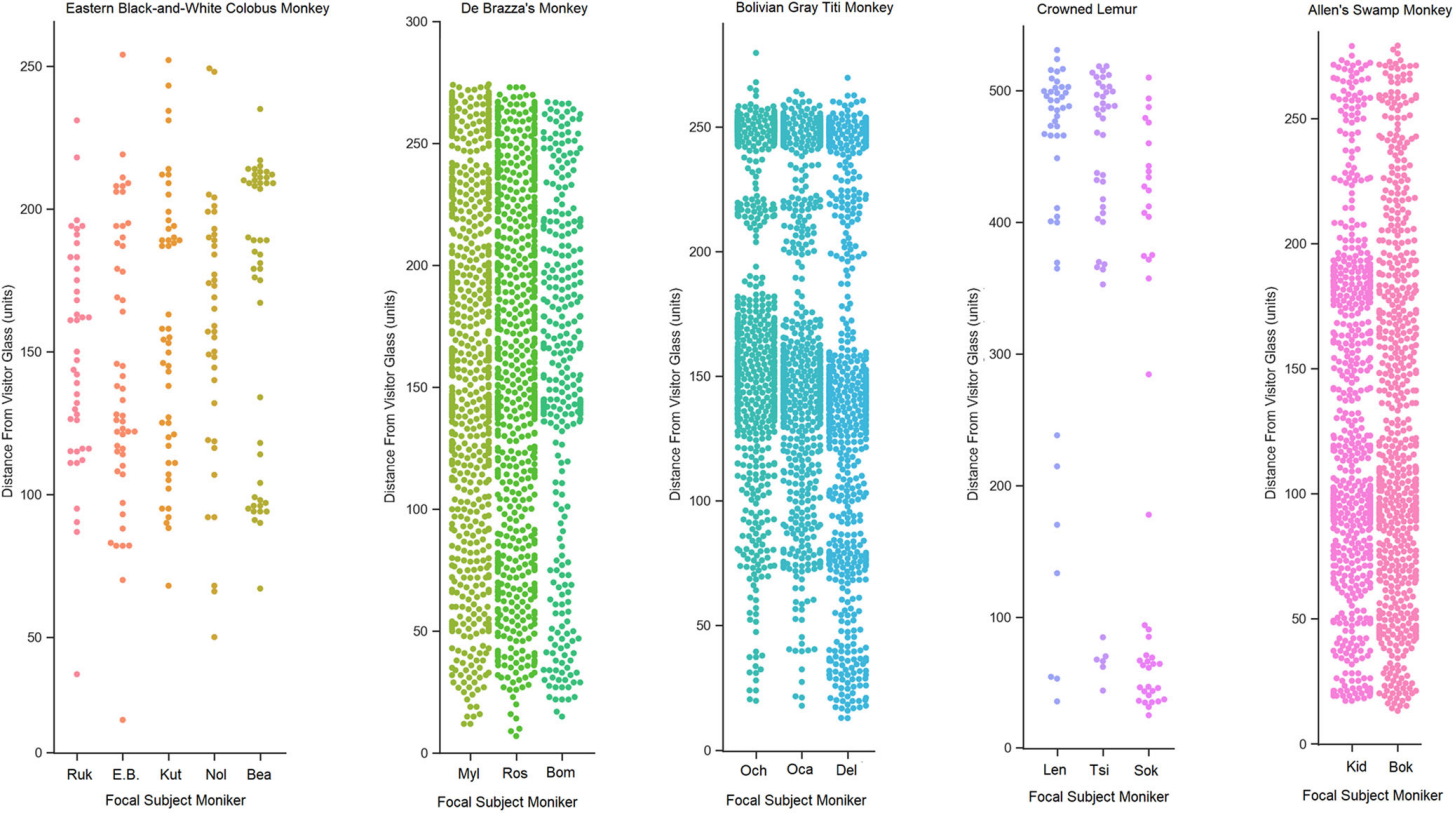
Graphs depicting all recorded distances for subjects. Each point represents a single instance of a focal subject's distance from visitor viewing glass as recorded during an observation period.

For this study, we focus on the location plotted in the first interval data (i.e., at 1 min) given the temporal proximity to the recording of the visitor count data which may fluctuate throughout the 10‐min session. When recording location of subjects in a given habitat, observers used the ZooMonitor app to plot primate location on a digital map presented on the touchscreen. This touch was recorded on a two‐dimensional 600 × 600 co‐ordinate pixel point system that generated the pixel units used for analysis here. These maps used to plot focal subject locations are shown in Figure 4. Table 2 defines the number of centimeters per pixel unit for each habitat. Habitat maps were not standardized as distances may carry different meanings across habitats depending on individual layout and features. Figure 5 illustrates the distribution of recorded distances from the visitor glass for each subject.

**Table 2:** Explanation of the approximate number of centimeters per unit for each habitat's 600 × 600 coordinate pixel point digital map in the ZooMonitor application.

Species	Habitat centimeters (cm) per unit
a Eastern black‐and‐white colobus monkey	1.720
DeBrazza's monkey	2.148
Bolivian gray titi monkey	1.430
Crowned lemur	0.978
a Allen's swamp monkey	1.720

^a^ Shared enclosure.

The behavior of the focal subject was recorded on 1‐min intervals using an instantaneous scan sampling approach. Once more, we consider behavioral data recorded on the first scan only. One ethogram was used for all primate species, and it included two behaviors expected to increase when welfare is compromised. These were pacing (Pomerantz et al., 2013) (defined as an animal moving along a fixed path without an apparent goal or function, tracing the same route at least three times), and self‐scratching (defined as an animal interacting with their body using hands or mouth in a slow deliberate movement across their fur or interacting with their body using hands or feet in rapid movements across their fur). Although self‐scratching occurs as a normal part of a primates' behavioral repertoire, we include it here because this behavior is known to increase with distress (Schino et al., 1991).

### 2.3 Data analysis

**Figure 6:**
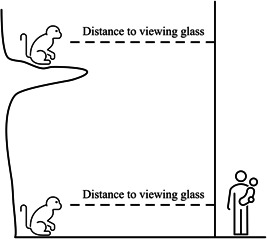
A diagram showing the calculation of primate distance to viewing glass as measured using 2‐dimensional linear distances. This figure demonstrates how the present analysis did not incorporate the differing distances associated with primates occupying varying heights.

Distances from viewing glass were calculated for each observation using the coordinates recorded in the ZooMonitor application. These coordinates exist on a two‐dimensional, horizontal plane, consisting of location values in the form of *x*‐*y* coordinates. The shortest distance from each animal location to the visitor glass was calculated in pixels (Figure 6). Note that this supplies a measure of the minimum two‐dimensional distance to the viewing glass. Visitors may be located along an exhibit boundary and animals may be at varying heights in their habitat.

A linear mixed model regression was conducted to examine space use as it relates to crowd size, with crowd size as the predictor variable for fixed effects and both species and individual as predictor variables for random effects (i.e., random intercept model). We do not aim to interpret differences between species in this study because four of the five species inhabited a unique exhibit and the role of species and exhibit could not be disentangled. Therefore, species is considered a random effect. Restricted maximum likelihood (REML) was used as the estimation method of model fit, and models were found not to violate assumptions of independence or homoskedasticity. This analysis was carried out using Python 3 (Van Rossum & Drake, 2011). In this analysis, visitor data were considered as categorical, binned predictor values with the following categories: 0, 1–5, 6–10, 11–15, 16–20, 21–25, 26–30, >30. We conducted a secondary analysis treating visitor presence as a dichotomous variable (present or absent) in a mixed‐effects logistic regression model with a binomial distribution and the same predictor variables as the linear mixed model.

To gain additional insight into the affective response of primates to the presence of visitors and provide more context for the interpretation of primates' space use, we analyzed whether the percent of scans in which subjects showed pacing or self‐scratching behaviors changed between sessions with visitors present and visitors absent. Given that the low occurrence of these behaviors generates a nonnormal distribution, we combined the two behaviors into one categorical behavioral indicator of welfare and investigated the difference using a nonparametric Wilcoxon signed ranks test.

This study was approved by the Lincoln Park Zoo Research Committee and adheres to the American Society of Primatologists (ASP) Principles for the Ethical Treatment of Nonhuman Primates.

## 3 RESULTS

**Figure 7:**
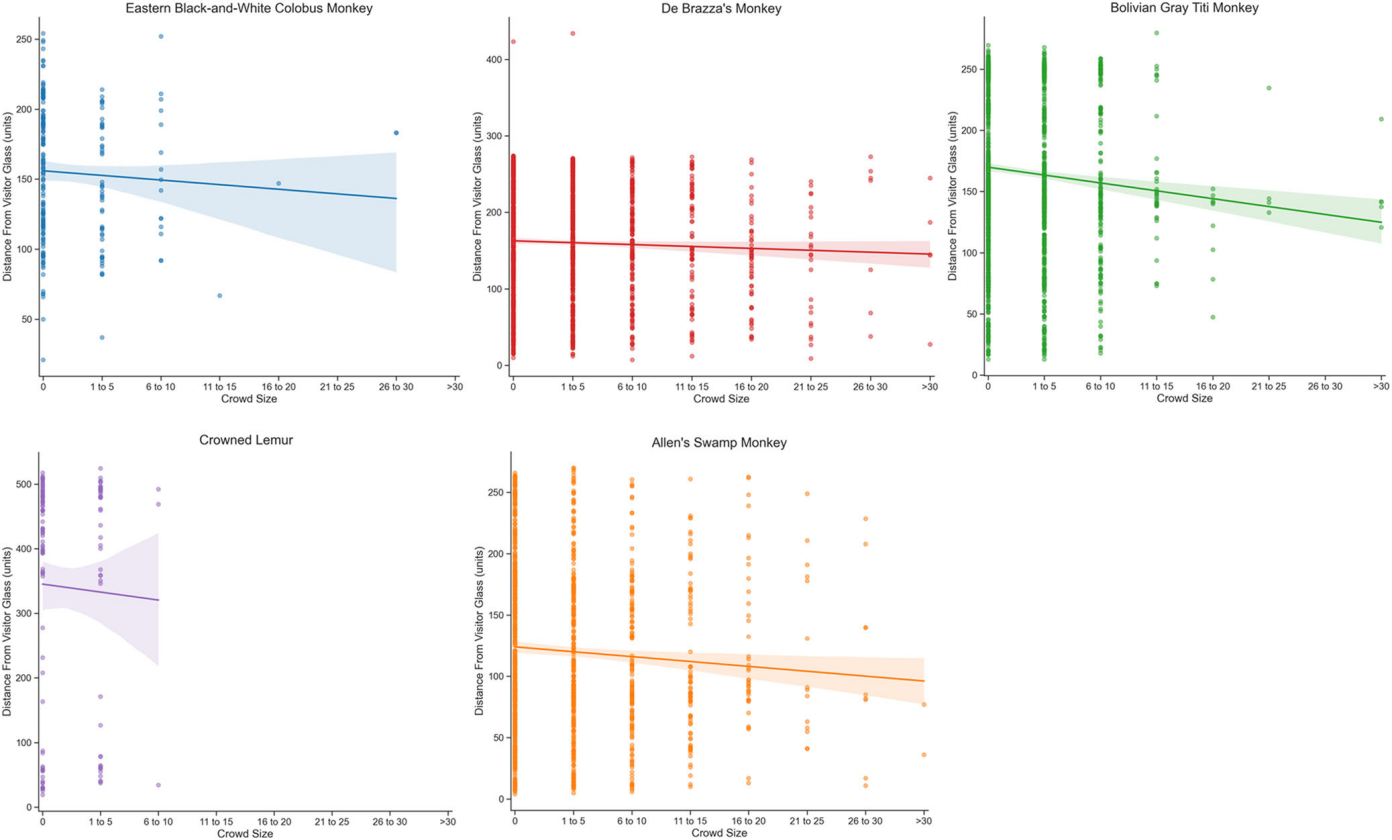
Results of the linear mixed model regression for each species. Distance from visitor viewing glass is represented in pixel units (*y*‐axis), and binned crowd size is shown on the *x*‐axis. Note that these data were collected across four different habitats, so comparison between panels should be taken with caution. The lack of any positive slopes indicates that no species increased distance from visitor glass as crowd sizes increased.

The number of observations obtained per individual and species is shown in Table 1. The results of the generalized linear mixed model revealed the number of visitors to be a significant predictor of primate distance from viewing glass (Table 3). As crowd sizes increased, primate distance from visitor glass slightly but significantly decreased (Figure 7), and the measures were negatively correlated. The results of the logistic regression, which considered the presence or complete absence of visitors, revealed the same effect of visitors: the presence of visitors was associated with a small but significant decrease in distance from the viewing glass (*β* = −9.800, SE = 3.770, 95% CI [−17.189, −2.411], *p* = 0.009). There was no difference in the behavioral indicators of welfare between sessions when visitors were present and absent (*N* = 13, *z* = 0.629, *p* = 0.529). The median percent of scans on which primates exhibited pacing or scratching during visitor‐absent observations was 2.16% (interquartile range = 4.60%); the median for visitor‐present observations was 1.97% (interquartile range = 5.95%).

**Table 3:** Summary of the generalized linear mixed model predicting primate distance from glass by number of visitors observing binned into standardized visitor counts.

Predictor	Coefficient	Standard error	*z*‐value	*p*‐value	Confidence interval [0.025 to 0.975]
Intercept	190.819	21.866	8.727	0.000	147.962 to 233.676
Crowd size (binned)	−4.901	1.874	−2.615	0.005	−8.573 to −1.228

## 4 DISCUSSION

In this study, we considered a relatively novel measure, distance from viewing window, to infer how visitors influence zoo animal welfare. Specifically, we measured whether zoo‐housed primates' distances from visitors changed in response to changing visitor crowd sizes. We used an ongoing long‐term data set and found a significant negative correlation indicating that the primates' distance from viewing glass decreased as the number of visitors increased. These data clearly demonstrate that the animals in the current study did not withdraw or retreat from visitor glass either when crowd sizes increased or when comparing between visitor presence and complete absence. We interpret this finding to suggest that visitors did not have a negative influence on the primates' affective state. We gain confidence in this interpretation when we consider that there was no change in the behavioral indicators of welfare while in the presence of visitors.

Visitors are a large part of the environment of animals living in zoos. This study used an objective measure of location within a given exhibit to infer the effect of visitors on the welfare of primates. If we had observed an increase in primate distances from the visitor glass with guest presence or increasing crow sizes, we would have inferred that visitors induced a negative affective state in the animals and having a negative influence on the animals' welfare status. Instead, we found a small decrease in distance from viewing glass with increasing crowd sizes, and on average when comparing times visitors were present with times visitors were absent, this distance decrease was approximately 15.7 cm. Because this decrease was very slight, we hesitate to interpret that as indicating a meaningful approach toward visitors but do feel confident that primates were not withdrawing from crowds.

Since we are suggesting others may want to employ this measure in future studies of animal welfare, it is worth considering how a more dramatic decrease in distance between animals and visitor viewing glass may be interpreted if found in future studies, as the interpretation of approach is less straightforward than interpretation of avoidance. Animals may be drawn toward stimuli that induce a negative mental state in some circumstances, as may have been shown in a recent study in which Japanese macaques were observed approaching visitors to engage in visitor‐directed aggression (Woods et al., 2019). To add additional complexity to approach interpretation, individual factors such as personality (e.g., Blaszczyk, 2017), personal characteristics and differences (e.g., Miller et al., 2021), or social rank (e.g., Woods et al., 2019) could also play a role. If a meaningful reduction in distance as a response to increasing visitor crowds is observed in future studies, considering behavioral data in more detail could prove helpful to interpret the impact on welfare.

The characteristics specific to these Lincoln Park Zoo habitats may also have played a role in the results of this study. All exhibits had glass barriers, and lower lighting in the visitor area than on exhibit. Results may be impacted by barrier type or visitor lighting levels as these impact the animals' perceptual and sensory experience in the habitat. Additionally, as described in Figure 3, small crowd sizes were observed more frequently than large crowd sizes. It is possible that patterns of visitor exposure could influence the behavioral patterns observed here.

There are several limitations to acknowledge in this study. First, this study did not consider exact distances from the primates in their exhibit to the closest visitor (Figure 6), nor did we consider the limited opportunities the animals had to move out of sight from visitors while on exhibit. An animal located above the viewing glass or in a space otherwise obstructed from visitor view may not be experiencing the same impact of visitor presence as one in direct sight. Additionally, due to a limitation in our data collection approach, we were not able to measure how often animals moved off exhibit in response to visitor presence. Instead, we were only able to consider where primates chose to be within their exhibits. A final limitation is that we did not have information about the behavior or activity levels of visitors. We believe it would be valuable to explore these measures in future work, given that some studies have found that visitor behavior may impact animal behavior independent of the number of visitors (Birke, 2002; Choo et al., 2011; Ramont et al., 2021b; Woods et al., 2019).

We invite other researchers looking to quantify welfare changes associated with visitor presence to consider incorporating space use, and specifically distance from visitors, in their studies. The approach described here is a relatively simple method of gathering data, and the location of animals and their distances from a point of interest in their environment is an objective measurement that can be used to consider animal comfort in many contexts (e.g., Ross et al., 2009; Sherwen et al., 2015; Wark et al., 2020; Williams et al., 2021a, 2021b). While welfare is complex, and an animal's environment and experiences variable, there is a benefit to simple measures that can provide insight into important topics, and we hope that future work will continue to investigate this measure.

## AUTHOR CONTRIBUTIONS

**Alexandria Cairo‐Evans**: Conceptualization (equal); formal analysis (equal); methodology (equal); visualization (equal); writing—original draft (equal); writing—review & editing (equal). **Natasha K. Wierzal**: Data curation (equal); methodology (equal); visualization (equal); writing—review & editing (equal). **Jason D. Wark**: Data curation (equal); methodology (equal); visualization (equal); writing—review & editing (equal). **Katherine A. Cronin**: Data curation (equal); methodology (equal); supervision (equal); writing—review & editing (equal).

## Data Availability

The data that support the findings of this study are available from the corresponding author upon reasonable request.

## References

[ajp23386-bib-0001] Binding, S. , Farmer, H. , Krusin, L. , & Cronin, K. (2020). Status of animal welfare research in zoos and aquariums: Where are we, where to next? Journal of Zoo and Aquarium, 8(3), 166–174. 10.19227/jzar.v8i3.505

[ajp23386-bib-0002] Birke, L. (2002). Effects of browse, human visitors and noise on the behaviour of captive orangutans. Animal Welfare, 11(2), 189–202.

[ajp23386-bib-0003] Blaszczyk, M. B. (2017). Boldness towards novel objects predicts predator inspection in wild vervet monkeys. Animal Behaviour, 123, 91–100. 10.1016/j.anbehav.2016.10.017

[ajp23386-bib-0004] Bonnie, K. E. , Ang, M. Y. , & Ross, S. R. (2016). Effects of crowd size on exhibit use by and behavior of chimpanzees (*Pan troglodytes*) and Western lowland gorillas (*Gorilla gorilla*) at a zoo. Applied Animal Behaviour Science, 178, 102–110. 10.1016/j.applanim.2016.03.003

[ajp23386-bib-0005] Boyle, S. A. , Berry, N. , Cayton, J. , Ferguson, S. , Gilgan, A. , Khan, A. , Lam, H. , Leavelle, S. , Mulder, I. , Myers, R. , & Owens, A. (2020). Widespread behavioral responses by mammals and fish to zoo visitors highlight differences between individual animals. Animals: An Open Access Journal from MDPI, 10(11), 2108. 10.3390/ani10112108 33203018 PMC7697811

[ajp23386-bib-0006] Choo, Y. , Todd, P. A. , & Li, D. (2011). Visitor effects on zoo orangutans in two novel, naturalistic enclosures. Applied Animal Behaviour Science, 133(1–2), 78–86. 10.1016/j.applanim.2011.05.007

[ajp23386-bib-0007] Claxton, A. M. (2011). The potential of the human–animal relationship as an environmental enrichment for the welfare of zoo‐housed animals. Applied Animal Behaviour Science, 133(1–2), 1–10. 10.1016/j.applanim.2011.03.002

[ajp23386-bib-0008] Collins, C. , Corkery, I. , Haigh, A. , McKeown, S. , Quirke, T. , & O'Riordan, R. (2017). The effects of environmental and visitor variables on the behavior of free‐ranging ring‐tailed lemurs (*Lemur catta*) in captivity. Zoo Biology, 36(4), 250–260. 10.1002/zoo.21370 28547779

[ajp23386-bib-0009] Costa, R. , Sousa, C. , & Llorente, M. (2018). Assessment of environmental enrichment for different primate species under low budget: A case study. Journal of Applied Animal Welfare Science, 21(2), 185–199. 10.1080/10888705.2017.1414606 29299937

[ajp23386-bib-0010] Das Gupta, M. , Das, A. , Sumy, M. C. , & MM, I. (2017). An explorative study on visitor's behaviour and their effect on the behaviour of primates at Chittagong zoo. Bangladesh Journal of Veterinary and Animal Sciences, 5(2), 24–32.

[ajp23386-bib-0011] Davey, G. (2007). Visitors' effects on the welfare of animals in the zoo: A review. Journal of Applied Animal Welfare Science, 10(2), 169–183. 10.1080/10888700701313595 17559323

[ajp23386-bib-0012] Fernandez, E. J. , Tamborski, M. A. , Pickens, S. R. , & Timberlake, W. (2009). Animal–visitor interactions in the modern zoo: Conflicts and interventions. Applied Animal Behaviour Science, 120(1–2), 1–8. 10.1016/j.applanim.2009.06.002

[ajp23386-bib-0013] Hansen, B. K. , Hopper, L. M. , Fultz, A. L. , & Ross, S. R. (2020). Understanding the behavior of sanctuary‐housed chimpanzees during public programs. Anthrozoös, 33(4), 481–495. 10.1080/08927936.2020.1771055

[ajp23386-bib-0014] Hashmi, A. , & Sullivan, M. (2020). The visitor effect in zoo‐housed apes: The variable effect on behaviour of visitor number and noise. Journal of Zoo and Aquarium, 8(4), 268–282. 10.19227/jzar.v8i4.523

[ajp23386-bib-0015] Hosey, G. , Ward, S. J. , Ferguson, A. , Jenkins, H. , & Hill, S. P. (2020). Zoo‐housed mammals do not avoid giving birth on weekends. Zoo Biology, 40(1), 3–8. 10.1002/zoo.21571 32956511

[ajp23386-bib-0016] Hutchings, K. , & Mitchell, H. (2003). A comparison of the behaviour of captive lemurs subjected to different causes of disturbance at Marwell Zoological Park, Proceedings of the 5th Annual Symposium on Zoo Research, Marwell Zoological Park (pp. 139–143). Federation of Zoological Gardens of Great Britain and Ireland.

[ajp23386-bib-0017] Jones, H. , McGregor, P. K. , Farmer, H. L. A. , & Baker, K. R. (2016). The influence of visitor interaction on the behavior of captive crowned lemurs (*Eulemur coronatus*) and implications for welfare. Zoo Biology, 35(3), 222–227. 10.1002/zoo.21291 27142724

[ajp23386-bib-0045] Learmonth, M. J. , Sherwen, S. , & Hemsworth, P. H. (2018). The effects of zoo visitors on Quokka (Setonix brachyurus) avoidance behavior in a walk‐through exhibit. Zoo Biology, 37(4), 223–228. 10.1002/zoo.21433 29992613

[ajp23386-bib-0019] Lincoln Park Zoo . (2020). *ZooMonitor (version 3.2.) [Mobile app]*. https://zoomonitor.org

[ajp23386-bib-0020] Mellor, D. J. , Beausoleil, N. J. , Littlewood, K. E. , McLean, A. N. , McGreevy, P. D. , Jones, B. , & Wilkins, C. (2020). The 2020 five domains model: Including human–animal interactions in assessments of animal welfare. Animals: An Open Access Journal from MDPI, 10(10), 1870. 10.3390/ani10101870 33066335 PMC7602120

[ajp23386-bib-0021] Mellor, D. J. , & Reid, C. S. W. (1994). Concepts of animal well‐being and predicting the impact of procedures on experimental animals. Improving the well‐being of animals in the research environment. Australian and New Zealand Council for the Care of Animals in Research and Teaching, 3–18.

[ajp23386-bib-0022] Miller, M. E. , Robinson, C. M. , & Margulis, S. W. (2021). Behavioral implications of the complete absence of guests on a zoo‐housed gorilla troop. Animals: An Open Access Journal from MDPI, 11(5), 1346. 10.3390/ani11051346 34065082 PMC8151611

[ajp23386-bib-0023] Pomerantz, O. , Meiri, S. , & Terkel, J. (2013). Socio‐ecological factors correlate with levels of stereotypic behavior in zoo‐housed primates. Behavioural Processes, 98, 85–91. 10.1016/j.beproc.2013.05.005 23694743

[ajp23386-bib-0024] Ramont, M. , Leahy, M. , & Cronin, K. A. (2021a). Domestic animal welfare at the zoo: The impact of an animal visitor interaction program on chickens. Animal Behavior and Cognition, 8, 1–14. 10.26451/abc.08.01.01.2021

[ajp23386-bib-0025] Ramont, M. , Leahy, M. , & Cronin, K. A. (2021b). The welfare of domestic goats (*Capra hircus*) in a zoo‐based animal‐visitor interaction program. Animal Behavior and Cognition, 8(4), 493–506. 10.26451/abc.08.04.04.2021

[ajp23386-bib-0026] Rose, P. E. , Scales, J. S. , & Brereton, J. E. (2020). Why the “visitor effect” is complicated. Unraveling individual animal, visitor number, and climatic influences on behavior, space use and interactions with keepers. A case study on captive hornbills. Frontiers in Veterinary Science, 7, 236. 32411742 10.3389/fvets.2020.00236 PMC7199352

[ajp23386-bib-0027] Ross, S. R. , Schapiro, S. J. , Hau, J. , & Lukas, K. E. (2009). Space use as an indicator of enclosure appropriateness: A novel measure of captive animal welfare. Applied Animal Behaviour Science, 121(1), 42–50. 10.1016/j.applanim.2009.08.007

[ajp23386-bib-0028] Schino, G. , Troisi, A. , Perretta, G. , & Monaco, V. (1991). Measuring anxiety in nonhuman primates: effect of lorazepam on macaque scratching. Pharmacology, Biochemistry and Behavior, 38(4), 889–891. 10.1016/0091-3057(91)90258-4 1871201

[ajp23386-bib-0029] Sherwen, S. L. , & Hemsworth, P. H. (2019). The visitor effect on zoo animals: Implications and opportunities for zoo animal welfare. Animals: An Open Access Journal from MDPI, 9(6), 366. 10.3390/ani9060366 31212968 PMC6617010

[ajp23386-bib-0030] Sherwen, S. L. , Hemsworth, P. H. , Butler, K. L. , Fanson, K. V. , & Matrath, M. J. L. (2015). Impacts of visitor number on kangaroos housed in free‐range exhibits. Zoo Biology, 34(4), 287–295. 10.1002/zoo.21226 26036594

[ajp23386-bib-0031] Sherwen, S. L. , Magrath, M. J. , Butler, K. L. , & Hemsworth, P. H. (2015). Little penguins, *Eudyptula minor*, show increased avoidance, aggression and vigilance in response to zoo visitors. Applied Animal Behaviour Science, 168, 71–76. 10.1016/j.applanim.2015.04.007

[ajp23386-bib-0032] Smith, K. N. , & Kuhar, C. W. (2010). Siamangs (*Hylobates syndactylus*) and white‐cheeked gibbons (*Hylobates leucogenys*) show few behavioral differences related to zoo attendance. Journal of Applied Animal Welfare Science, 13(2), 154–163. 10.1080/10888700903579895 20349380

[ajp23386-bib-0033] Todd, P. A. , Macdonald, C. , & Coleman, D. (2007). Visitor‐associated variation in captive Diana monkey (*Cercopithecus diana diana*) behaviour. Applied Animal Behaviour Science, 107(1–2), 162–165. 10.1016/j.applanim.2006.09.010

[ajp23386-bib-0034] Van Rossum, G. , & Drake, F. L. (2011). The python language reference manual. Network Theory Ltd.

[ajp23386-bib-0036] Wark, J. D. , Cronin, K. A. , Niemann, T. , Shender, M. A. , Horrigan, A. , Kao, A. , & Ross, M. R. (2019). Monitoring the behavior and habitat use of animals to enhance welfare using the ZooMonitor app. Animal Behavior and Cognition, 6(3), 158–167. 10.26451/abc.06.03.01.2019

[ajp23386-bib-0037] Wark, J. D. , Wierzal, N. K. , & Cronin, K. A. (2020). Mapping shade availability and use in zoo environments: A tool for evaluating thermal comfort. Animals: An Open Access Journal from MDPI, 10(7), 1189. 10.3390/ani10071189 32674340 PMC7401555

[ajp23386-bib-0038] Watters, J. V. , Krebs, B. L. , & Eschmann, C. L. (2021). Assessing animal welfare with behavior: Onward with caution. Journal of Zoological and Botanical Gardens, 2(1), 75–87. 10.3390/jzbg2010006

[ajp23386-bib-0039] Williams, E. , Carter, A. , Rendle, J. , & Ward, S. J. (2021a). Understanding impacts of zoo visitors: Quantifying behavioural changes of two popular zoo species during COVID‐19 closures. Applied Animal Behaviour Science, 236, 105253. 10.1016/j.applanim.2021.105253 36540094 PMC9755048

[ajp23386-bib-0040] Williams, E. , Carter, A. , Rendle, J. , & Ward, S. J. (2021b). Impacts of COVID‐19 on animals in zoos: A longitudinal multi‐species analysis. Journal of Zoological and Botanical Gardens, 2(2), 130–145. 10.3390/jzbg2020010

[ajp23386-bib-0041] Wood, W. (1998). Interactions among environmental enrichment, viewing crowds, and zoo chimpanzees (*Pan troglodytes)* . Zoo Biology, 17(3), 211–230. 10.1002/(SICI)1098-2361(1998)17:3<211::AID-ZOO5>3.0.CO;2-C

[ajp23386-bib-0042] Woods, J. M. , Ross, S. R. , & Cronin, K. A. (2019). The social rank of zoo‐housed Japanese macaques is a predictor of visitor‐directed aggression. Animals: An Open Access Journal from MDPI, 9(6), 316. 10.3390/ani9060316 31159468 PMC6617189

[ajp23386-bib-0043] Ydenberg, R. C. , & Dill, L. M. (1986). The economics of fleeing from predators. Advances in the Study of Behavior, 16, 229–249. 10.1016/S0065-3454(08)60192-8

